# A giant urethral stone with urethrocutaneous fistula: A case report

**DOI:** 10.1016/j.eucr.2024.102848

**Published:** 2024-09-11

**Authors:** Henry Pramana, Jupiter Sibarani

**Affiliations:** Department of Urology, Padjadjaran University/Hasan Sadikin General Hospital, Bandung, Indonesia

**Keywords:** Urethrocutaneous fistula, Giant urethral stone, Multiple urethrolithiasis

## Abstract

Calculi in the urethra are uncommon, and urethral calculi causing urethrocutaneous fistula are extremely rare. A 56 years-old man with history of urine passage from his scrotal area for a month. During physical examination, we found a multiple fistula in scrotal area and revealed by the ultrasound. Abdominal x-ray suggests a vesicolithiasis and multiple urethrolithiasis. To date, there are 14 cases of giant urethral stone with and without urethrocutaneous fistula reported in literature worldwide for the last 14 years. It was related to patients that delayed to seek a medical attention from his symptoms.

## 1 Introduction

Calculi in the urethra are uncommon, representing only 1–2% of all calculi in the genito-urinary tract, and urethral calculi causing urethrocutaneous fistula are extremely rare.^1^ Huge urethral stone usually present in prostatic urethra, with uncommonly present in anterior urethra.^1^ Native urethral calculi (formed in the urethra) related to a urethral fistula with complications includes trauma and infection compared to migratory stones (those descent from the upper urinary tract). The main reason of urethral fistula may be associated with late treatment.^2^ Here we report cases of spontaneous fistula caused by urethral stricture with giant posterior urethral stones and multiple anterior urethral stone with peri penile abscess and diverticula.

## 2 Case presentation

**Fig. 1:**
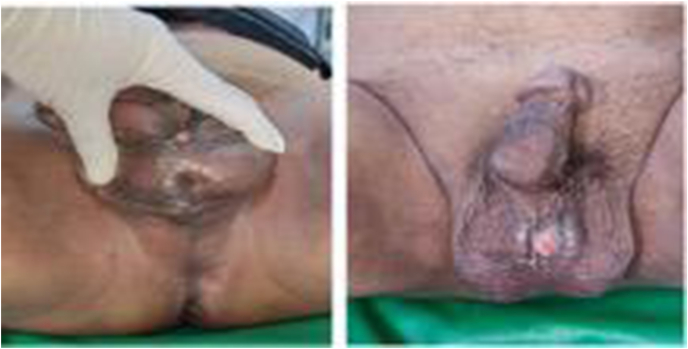
Multiple fistulae at physical examination in scrotal region.

**Fig. 2:**
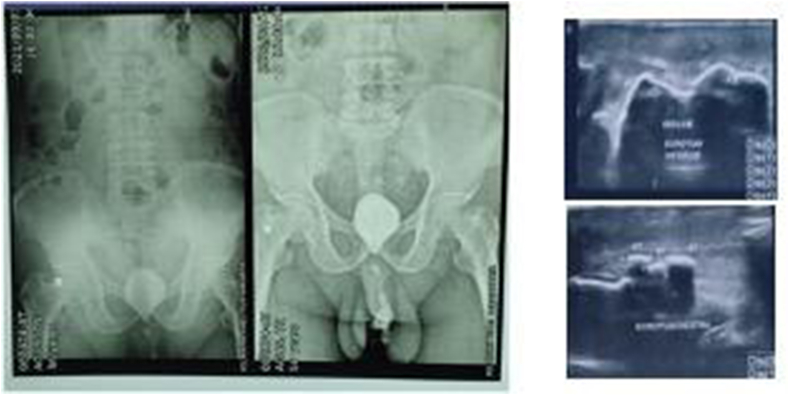
Abdominal X-ray and scrotal ultrasound showed suggestive of vesicolithiasis and multiple urethral stones.

**Fig. 3:**
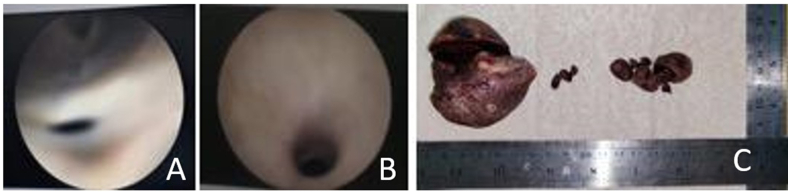
(A) Urethroscopy revealed fistula at penile region and (B) stricture at the bulbous urethra. (C) A giant posterior urethral stones and multiple anterior urethral stones successfully evacuated.

A 56 years-old man came our clinic with history of passage of urine from his scrotal area 1 month's duration every time his urination. He had a history of difficulty urination, straining, weak stream urine, splitting urine and dysuria. He had also discharge of purulent fluid from his penis a months later. During physical examination, we found a multiple fistula in scrotal area (Fig. 1) with normal digital rectal examination. From abdominal x-ray we found an opaque concremen on the pelvis and multiple opaque lession in the penile area suggestive of vesicolithiasis and multiple urethrolithiasis. From scrotal ultrasound reveal multiple hyperechoic lesions with posterior acoustic shadow in scrotum (Fig. 2). We decided to perform urethroscopy, there are fistula at anterior urethra, and stricture at bulbosa (Fig. 3A and B), we performed stricture release with urethral dilator, then we found stone at prostatic urethra. We performed midline incision and open bladder, we found narrow bladder neck and we did not find a bladder stones, and then retropubic urethrotomy (millin procedure) to take out the stone was performed, we found 50 × 60mm calculi (Fig. 3C). Then the urethra was sutured with vicryl 3.0, we also performed urethral diverticula excision and periurethral abscess debridement. Patient was inserted 16fr cystostomy catheter, 24fr 3-way foley catheter and retropubic drain. We diagnosed the patient with multiple urethral stone + urethrocutaneous fistula + periurethral abscess + anterior urethral stricture + urethra diverticula. Two weeks after operation we took out the catheter and the patient can void spontaneously with no urine leakage.

## 3 Discussion

Urethral calculi amount to 0.3 %–1 % of all stone disease, and obstructing urethral calculus is a very rare presentation of lower urinary tract urolithiasis. The clinical presentation depends on the type and location of the stone. Patients with migratory calculi usually have a history of stone disease, previous surgeries for stone or instrumentation of lower tract, a vague history of flank pain in the preceding 1 or 2 weeks, suggesting origin from the upper tracts, and lower urinary tract symptoms.^3^ Posterior urethral stones typically have perineal or rectal pain, whereas those in the pendulous urethra have pain at the penile tip. Patients with primary urethral calculi or diverticular calculi have more insidious symptoms of persistent pain during voiding, obstructive lower urinary tract symptoms, chronic pelvic pain, or recurrent urinary tract infections. Women tend to report increased urinary frequency and occasional incontinence.^4^ Those who are able to void with minimal symptoms and those with urethral diverticula often seek medical attention late with a long history, often up to many years. Prolonged delays have a more complicated presentation with larger calculi and urethra-cutaneous or urethra-rectal fistulas.^5^

We collect 8 literatures related to large or giant urethral stone with or without urethrocutaneous fistula from the last 14 years published articles as the incidence was very rare. Online searching was performed from online databases includes PubMed, ScienceDirect, and Google Scholar using search terms of large of giant urethral stone, urethral calculus, and urethrocutaneous fistula. Based on these 8 literatures, the urethral stones were considered as large or giant when the size is ≥ 2 cm. Five of the cases reported with urethrocutaneous fistula and one cases with vesicocutaneous fistula. Location of stones were varied in those cases includes anterior urethra, penile urethra, posterior urethra, and prostatic urethra. Obstructive lower urinary tract symptoms and acute urinary retention were the main reason of those patients to be admitted to emergency department. The smallest of giant urethral stone reported was a stone of 3 cm in size. Other studies mentioned giant urethral calculi in size of 6 × 3 cm, 5.9 × 3.2 × 2.8 cm, 6.5 × 2.7 × 2.5 cm.

An important cause of a urethral fstula may be related to delayed treatment. They may have occurred as a result of a penile-scrotum abscess. Patients with primary urethral stones were usually asymptomatic or had chronic voiding problems. Treatment of urethral calculi depends on the location within the urethra and the distance from the internal or the external urethral meatus, stone characteristics, the ability of the stone to get pushed into the bladder, and associated structural abnormalities of the urethra, if any. In our study, the case presentation is also similar with Kaplan et al. which reported giant prostatic urethral calculus with urethrocutaneous fistula.^5^ When complicated by urethral stricture or urethra-cutaneous fistula, simultaneous or staged repair should be undertaken. Stones within the diverticula are usually treated with incision of the diverticulum and stone extraction. Diverticulectomy and urethral repair is usually required and can be done either in a same sitting or in a staged fashion.^5^

## 4 Conclusion

There are some literatures reported that giant urethral stones associated with the urethrocutaneous fistula. The complication of giant urethral stones is urethrocutaneous fistula. Most of this case was related to patient that delayed to seek medical attention for his symptoms. Successful treatment is mostly done by excision of the fistulous tract, retrieval of the urethral stones, and debridement of pus.

## Consent

I hereby declare that ethical clearance was not required for this research project as it did not involve human or animal experimentation or any sensitive data. The nature of the study and the methods employed strictly adhered to ethical standards and guidelines. Consequently, no ethical clearance was sought or obtained for the completion of this research. Consent was obtained from the patient for publication of this article.

## CRediT authorship contribution statement

**Henry Pramana:** Writing – review & editing, Writing – original draft, Investigation, Formal analysis, Data curation, Conceptualization. **Jupiter Sibarani:** Validation, Supervision, Methodology, Conceptualization.

## Declaration of competing interest

The authors declare that they have no conflicts of interest.
